# Supplementary material for: An Algorithm Framework for Drug-Induced Liver Injury Prediction Based on Genetic Algorithm and Ensemble Learning
Source: Molecules. 2022 May 12;27(10):3112. doi: 10.3390/molecules27103112 (PMC9147181; doi:10.3390/molecules27103112)
Supplement: Supplementary file 1 [file molecules-27-03112-s001.zip › molecules-1704040-supplementary.pdf]

# Supplementary Materials:

## 1. Supplementary Table S1.

The molecular fingerprints involved in this calculation are described below, where Rdkit is a continuous variable and other fingerprints are discrete variables.

Table S1. Molecular fingerprints information

| Molecules Fingerprints | Size | Description | Source |
|---|---|---|---|
| Daylight | 2048 | Daylight is a 2048-length vector that encodes path-based substructures. A multi-layer perceptron is then applied on top of the vector. | Rdkit |
| ECFP2 | 2048 | ECFP2 is a 2048-length bit vector, which represents the neighborhood environment of each atom using the extended connectivity fingerprint encoding a circular substructure of diameter 2 bonds. An individual bit has no definite meaning. | Rdkit |
| ECFP4 | 2048 | ECFP2 is a 2048-length bit vector, which represents the neighborhood environment of each atom using the extended connectivity fingerprint encoding a circular substructure of diameter 4 bonds. An individual bit has no definite meaning. | Rdkit |
| ECFP6 | 2048 | ECFP6 is a 2048-length bits vector, which represents the neighborhood environment of each atom using the extended connectivity fingerprint encoding a circular substructure of diameter 6 bonds. An individual bit has no definite meaning. | Rdkit |
| MACCS | 168 | MACCS is a 168-length bits vector, which is substructure key-based fingerprints representing the presence of certain substructures or fragments from a given list of structural keys in the compound. | Rdkit |
| Pubchem | 881 | PubchemFP is an 881-length bit vector, where each bit corresponds to hand-crafted important substructures. A multi-layer perceptron is then applied on top of the vector. | Rdkit |
| Rdkit2D | 200 | Rdkit2D is a 200-length vector that describes the global pharmacophore descriptor. It is normalized to make the range of the features in the same scale using cumulative density function fit given a sample of the molecules. | https: //github.com/bp-kelley/descriptastorus |
| MorganFP | 1024 | MorganFP is a 1024-length bits vector that encodes circular radius-2 substructures. A multi-layer perceptron is then applied to the binary fingerprint vector. | Rdkit |

2. Supplementary Table S2.

The performance of DILI predition with different machine learning models and fingerprints in pre-experiment.

Table S2.1 ACC of the Pre-trained performation with different Fingerprints

| Fingerprints | RandomForest | SVC | Xgboost | GBDT | Adaboost | LR | DT |
|---|---|---|---|---|---|---|---|
| Daylight | 0.727 | 0.727 | 0.717 | 0.709 | 0.657 | 0.67 | 0.673 |
| ECFP2 | 0.752 | 0.68 | 0.731 | 0.714 | 0.667 | 0.691 | 0.689 |
| ECFP4 | 0.752 | 0.655 | 0.731 | 0.713 | 0.667 | 0.709 | 0.678 |
| ECFP6 | 0.753 | 0.612 | 0.714 | 0.704 | 0.652 | 0.69 | 0.66 |
| MACCS | 0.744 | 0.675 | 0.729 | 0.706 | 0.643 | 0.682 | 0.677 |
| Pubchem | 0.742 | 0.662 | 0.721 | 0.723 | 0.674 | 0.694 | 0.675 |
| Rdkit2D | **0.755** | 0.68 | 0.74 | 0.739 | 0.674 | 0.691 | 0.661 |
| MorganFP | 0.744 | 0.657 | 0.717 | 0.724 | 0.653 | 0.675 | 0.678 |

Table S2.2 F1-score of the Pre-trained performation with different Fingerprints

| Fingerprints | RandomForest | SVC | Xgboost | GBDT | Adaboost | LR | DT |
|---|---|---|---|---|---|---|---|
| Daylight | 0.726 | 0.726 | 0.716 | 0.708 | 0.656 | 0.669 | 0.672 |
| ECFP2 | 0.751 | 0.67 | 0.73 | 0.713 | 0.666 | 0.691 | 0.689 |
| ECFP4 | 0.751 | 0.623 | 0.73 | 0.712 | 0.665 | 0.708 | 0.678 |
| ECFP6 | 0.752 | 0.55 | 0.713 | 0.703 | 0.65 | 0.69 | 0.66 |
| MACCS | 0.743 | 0.673 | 0.729 | 0.706 | 0.642 | 0.681 | 0.676 |
| Pubchem | 0.741 | 0.662 | 0.721 | 0.722 | 0.673 | 0.693 | 0.675 |
| Rdkit2D | **0.753** | 0.677 | 0.739 | 0.7237 | 0.674 | 0.69 | 0.66 |
| MorganFP | 0.743 | 0.626 | 0.717 | 0.723 | 0.652 | 0.674 | 0.677 |

Table S2.3 AUC of the Pre-trained performation with different Fingerprints

| Fingerprints | RandomForest | SVC | Xgboost | GBDT | Adaboost | LR | DT |
|---|---|---|---|---|---|---|---|
| Daylight | 0.744 | 0.695 | 0.718 | 0.71 | 0.658 | 0.67 | 0.674 |
| ECFP2 | 0.752 | 0.679 | 0.731 | 0.714 | 0.667 | 0.692 | 0.69 |
| ECFP4 | 0.753 | 0.65 | 0.732 | 0.713 | 0.666 | 0.708 | 0.679 |
| ECFP6 | 0.753 | 0.608 | 0.714 | 0.704 | 0.651 | 0.691 | 0.661 |
| MACCS | 0.743 | 0.674 | 0.729 | 0.707 | 0.644 | 0.683 | 0.677 |
| Pubchem | 0.742 | 0.662 | 0.721 | 0.723 | 0.675 | 0.694 | 0.676 |
| Rdkit2D | **0.805** | 0.744 | 0.739 | 0.739 | 0.675 | 0.691 | 0.661 |
| MorganFP | 0.793 | 0.751 | 0.718 | 0.724 | 0.654 | 0.675 | 0.678 |
